# Molecular markers and molecular basis of plant type related traits in maize

**DOI:** 10.3389/fgene.2024.1487700

**Published:** 2024-11-01

**Authors:** Xinghua Zhao, Changbiao Wang, Jiang Liu, Bin Han, Jinling Huang

**Affiliations:** ^1^ College of Agriculture, Shanxi Agricultural University, Taigu, Shanxi, China; ^2^ College of Life Sciences, Shanxi Agricultural University, Taigu, Shanxi, China

**Keywords:** maize, plant type traits, SNP, molecular markers, molecular basis

## Abstract

Maize, belonging to the Poaceae family and the *Zea* L. genus, stands as an excellent food crop. The plant type has a significant impact on crop growth, photosynthesis, lodging resistance, planting density, and final yield. In this study, 160 maize inbred lines were selected as experimental materials to conduct molecular markers research on maize plant type traits through the measurement of plant type-related traits, population structure, and genome-wide association analysis. The phenotypic data revealed differences in plant type-related traits among maize inbred lines grown in the Xinzhou and Jinzhong regions. The frequency distribution of plant height, ear height, spindle length of tassel, and first-order branch number of tassel traits in the 160 maize inbred lines previously studied generally conformed to a normal distribution. We identified 42,240 high-quality single nucleotide polymorphisms (SNPs) using the Affymetrix Axiom chip. The 160 maize inbred lines were categorized into six subgroups, each exhibiting an average gene diversity of 0.356 and an average polymorphism information content of 0.245. We identified 9, 23, 18, 8 and 32 loci that were significantly associated with first-order branch number of tassel, spindle length of tassel, ear height, plant height, and ear height/plant height ratio, respectively. At the same time, 6, 22, 14, 2, and 37 genes were identified as significantly associated with first-order branch number of tassel, spindle length of tassel, ear height, plant height, and ear height/plant height ratio, respectively. This study comprehensively delved into the genetic information of maize plant type-related traits, offering valuable genetic resources and a solid theoretical foundation for the breeding of novel maize varieties with optimized plant types.

## Introduction

Plant type-related traits in maize are one of the important factors affecting plant photosynthesis, growth and development, and grain yield (Tokatlidis, 2022; Ahn et al., 2023). An ideotype can make full use of the suitable growth environment. Plant height, ear height, and tassel traits, as the main plant type traits, can affect plant canopy structure, leaf distribution, photosynthetic efficiency, biological yield, and lodging resistance, and are important components of ideotype construction (Ahn et al., 2023). Proper reduction of ear height and plant height can enhance the harvest index and economic yield of maize, increase the efficiency of light energy and nitrogen utilization, and improve stress and lodging resistance (Khush, 2001; Weng et al., 2011). Recent years have witnessed a surge in research interest on maize plant type-related traits, including plant height, ear height, spindle length of tassel, and the number of tassel branches (Lin et al., 2007; Wang and Wang, 2015).

The rapid advancement of modern molecular biology techniques, including gene cloning, has transitioned genetic markers from cytological, morphological, and biochemical markers to molecular markers at the DNA level (Frascaroli et al., 2013). As gene chip and identification technologies continue to advance rapidly, the accuracy and throughput of single nucleotide polymorphisms (SNP) genotyping will steadily increase, while costs will decline. The SNP chip is a method of extracting and fluorescently labeling the nucleic acid sequence to be detected, hybridizing it with a probe with a specific base sequence, and finally measuring the base type of the sequence to be detected based on the type and intensity of the laser-scanned fluorescence signal (Lu et al., 2009). SNP markers assume a pivotal role in unraveling genetic structures and variations, gene discovery, and functional validation in organisms (Natesan et al., 2020). Currently, the application of SNP extends beyond basic study, finding widespread use in applied study as well (Ganal et al., 2009; Ganal et al., 2012).

Genome-wide association study (GWAS), a novel genetic technology, uses SNP marker loci to correlate with plant phenotypes, effectively analyzing the genetic mechanisms of complex quantitative traits in plants and promoting plant breeding work. In maize, GWAS technology was used to analyze the genetic variation of the *ZmVPP1* gene and confirm the genetic basis of the *ZmVPP1* gene’s response to drought stress (Wang et al., 2016). GWAS have identified new candidate loci or genes that affect maize stalk strength (Xu et al., 2023). Meanwhile, the joint linkage map and GWAS reveal a wide range of genetic loci that regulate the size of male inflorescences in maize (Wu et al., 2016). Furthermore, an abundance of research endeavors have adopted the application of GWAS technology to meticulously dissect and analyze the pertinent research pertaining to quantitative traits in maize (Lin et al., 2022; Wang et al., 2023; Sahito et al., 2024).

Maize inbred lines, through extensive breeding practices, have accumulated a wealth of genetic variations, resulting in a high degree of genetic diversity (Liu et al., 2003; Azmach et al., 2013). In recent years, maize research has mainly focused on yield, quality, and stress resistance, and has achieved remarkable results. However, there has been relatively little research on plant type-related traits (Flores-Hernández et al., 2023; Gao et al., 2024; Tian et al., 2024). This research utilized maize inbred line populations as the experimental materials to conduct a comprehensive study encompassing the measurement of plant type-related traits, Affymetrix Axiom^®^ chip genotyping, analysis of genetic diversity, and genome-wide association studies. In this study, we had successfully identified high-quality SNPs in maize inbred lines, and furthermore, we had uncovered pivotal genes that regulated plant type traits, includingplant height, ear height, ratio of ear height to plant height, spindle length of tassel and first-order branch number of tassel. This study undertakes a genome-wide scanning of plant type-related traits, thereby offering genetic resources and a solid theoretical foundation for the breeding of novel maize varieties characterized by ideotype.

## Materials and methods

### Plant materials

A total of 160 maize inbred line material populations were selected for the experiment. All materials were provided by the Maize Breeding Research Group of the College of Life Sciences at Shanxi Agricultural University (Biotechnology Research Center of Shanxi Academy of Agricultural Sciences). The names/sources of the materials were shown in Supplementary Table S1. 160 maize inbred lines were planted in Jinzhong City (111°25′∼114°05′E, 36°40′∼38°06′N) and Xinzhou City (110°53'∼113°58′E, 38°6′∼39°40′N). The field experiment was designed as a completely randomized block experiment with three replicates. Each material was sown in a single row with a row length of 5 m, a width of 2 m, and a plant spacing of 0.3 × 0.5 m. The plants were left in the holes, and the field management was conducted using conventional management methods. The main agronomic traits of the plant type were investigated.

### Measurement of maize plant type-related traits

Ten maize plants with normal growth 20 days after pollination were selected for plant type-related trait measurements in each cultivation experiment plot. The height from the ground surface to the top of the plant’s tassel, measured with a ruler (Unit: cm), was defined as the plant height. While measuring the plant height, the height from the ground surface to the uppermost ear bearing node was considered as the ear height. We also calculated the ratio of ear height to plant height. The length from the lowest branch of the main axis of the tassel to the top of the main axis of the tassel was measured and defined as the spindle length of tassel. The first-order branch number of tassel was determined as the count of first-order lateral branches present during the peak period of pollen shedding in the maize plant.

### Affymetrix axiom chip analysis of maize inbred lines

We extracted genomic DNA from 160 maize inbred lines using the CTAB method (Saghai-Maroof et al., 1984; Devos and Gale, 1992). The chip DNA library was conducted according to the Affymetrix Axiom data requirements protocol (Ganal et al., 2011; Ganal et al., 2012). Denaturing hybridization was performed using the Affymetrix GeneTitan system (Ganal et al., 2011; Ganal et al., 2012). After denaturation, the sample and Hyb Tray were placed on a 48°C metal block for operation. The denatured sample was added to the Hyb Tray and hybridized for 23.5–24 h at a designated location according to the requirements of the Affymetrix GeneTitan system. Finally, staining, cleaning, and scanning were performed in the study. The original data CELL file was classified based on several indicators: the percentage of successful loci in the sample Call relative to the total number of loci, Heterozygous Strength Offset, Fisher’s Linear Discriminant, Homozygote Ratio Offset, the number of minor alleles, and the minimum distance between the cluster centers of homozygous and heterozygous loci in HomFLD. We conducted a thorough analysis of the raw data quality, encompassing the loci deletion rate, minor allele frequency (MAF), sample deletion rate, and verification of hardy-weinberg equilibrium (HWE). Samples and loci with a high rate of missing typing and loci with a low MAF were filtered out in subsequent studies, as well as typing results that deviated from the HWE locus in normal controls (Tzeng and Sullivan, 2010).

### Analysis of genetic diversity in maize inbred line population

The quality control of the SNPs obtained from 160 maize inbred lines was performed, and the loci with a typing success rate of less than 90% and a MAF of less than 0.05 were filtered out. The distribution of SNPs on chromosomes was obtained using high-quality genomes of maize (NCBI RefSeq assembly: GCF_902167145.1). Population structure analysis of 160 maize inbred lines was conducted using Admixture software, which employs a likelihood nested model (Alexander et al., 2009). The optimal number of groups was determined as the number of clusters for samples with the minimum cross-validation error value (Alexander et al., 2009). The genetic relationships among 160 maize inbred lines were calculated using SPAGeDi software (Hardy and Vekemans, 2002; Kim and Beavis, 2017), and the sample genetic relationship heatmap was plotted using R language’s pheatmap. The phylogenetic relationships of 160 maize inbred lines were constructed using the maximum likelihood method with the RAxML (v.8) software (Stamatakis, 2014).

### Genome-wide association analysis of plant type-related traits in maize

To uncover the associations between maize plant type-related traits and molecular markers, a genome-wide scan of high-density molecular markers was performed on a population of maize inbred lines, resulting in the identification of significant SNPs associated with these traits. To analyze the population structure of the samples, we utilized the admixture software, assuming a range of 1–10 clusters (K values) for the samples and conducted clustering accordingly. We then performed cross-validation on the clustering outcomes to identify the optimal number of clusters, which was determined by the trough of the cross-validation error rate. We performed association analysis using both the Q + K and P + K models using the population structure data matrix, kinship matrix, and principal component (PC) scores as covariates, along with SNP markers and phenotypic data. We used the GAPIT3 software to perform genome-wide association analysis on five phenotypes, including first-order branch number of tassel, spindle length of tassel, ear height/plant height ratio, plant height and ear height (Wang and Zhang, 2021). To reduce false positives in association analysis results by combining population structure information and kinship matrix of samples, seven models including GLM, MLM, MLMLM, CMLM, ECMLM, SUPER, and Blink were used to perform association analysis for each trait. The model was then evaluated and selected based on the Quartile-Quartile plot (Q-Q plot) (Dehghan, 2018). We used the “qqman” package of R language to draw Q-Q and manhattan plots from GWAS results (Turner, 2014).

### Identification and functional annotation of genes significantly associated with maize plant type-related traits

We selected the candidate regions 100 kb upstream and downstream of the significant SNPs, and simultaneously extracted all genes within the candidate regions as significant associated genes. The TBtool software was used to compare and extract significantly associated genes between Jinzhong and Xinzhou (Chen et al., 2020). Functional annotation of associated gene expression proteins was performed using Non-Redundant Protein Sequence Database (https://www.biostars.org/). Additionally, proteins were annotated in a hierarchical manner using the UniProt database. Initially, annotations were derived from the TrEMBL computer-predicted database, which were then refined through the manually curated Swiss-Prot database of protein sequences (UniProt-Consortium, 2023). The Pfam database was used to identify the structural domains and functional information of the associated gene expression proteins (Mistry et al., 2021). We conducted functional annotation and pathway enrichment of significantly associated genes with plant type traits in maize based on the Gene Ontology (GO) and Kyoto Encyclopedia of Genes and Genomes (KEGG) databases (Gene-Ontology-Consortium, 2019; Jin et al., 2023).

## Results

### Phenotypic characteristics related to plant type of maize inbred lines

**FIGURE 1:**
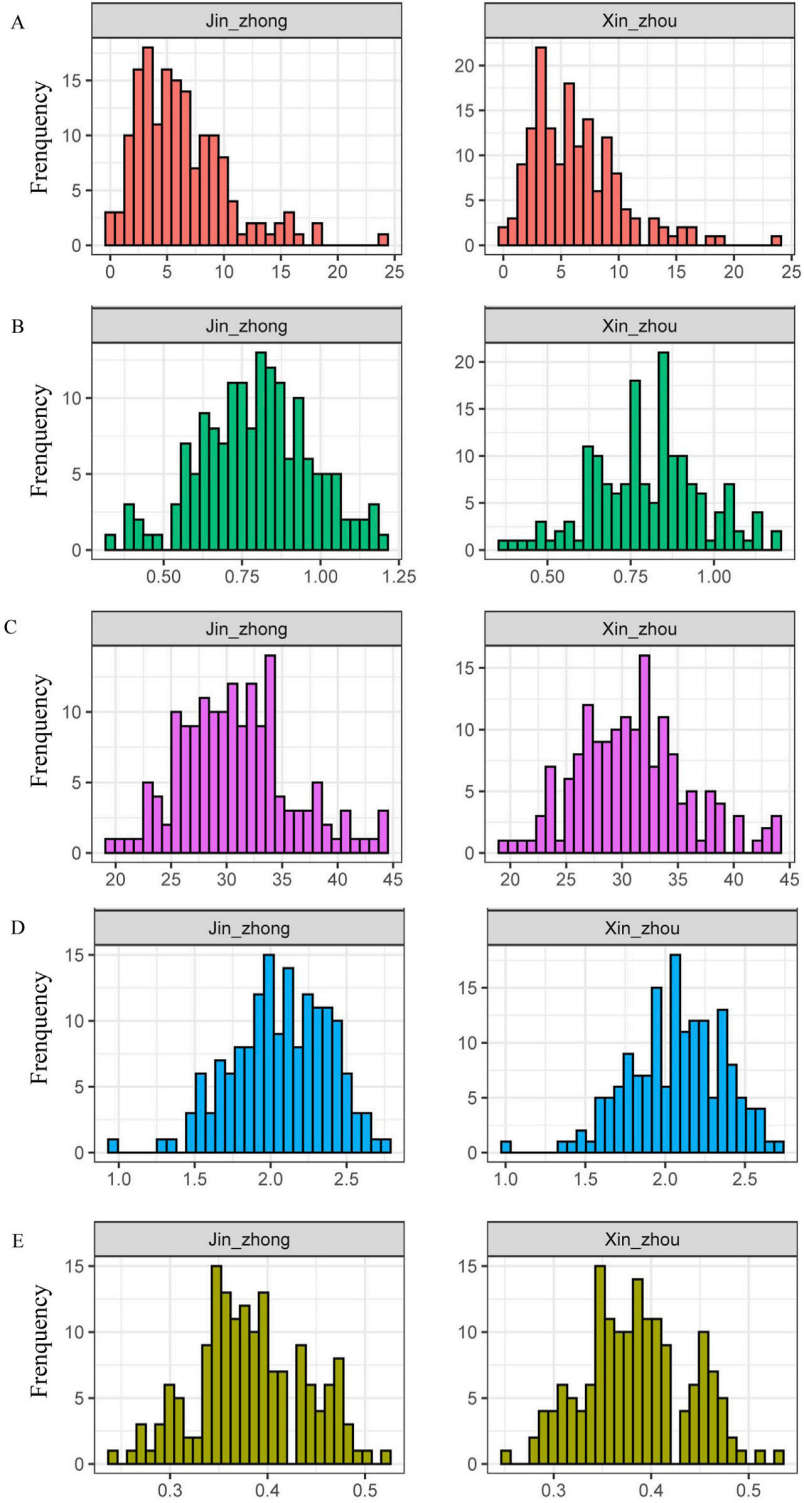
Phenotypic characteristics related to plant type of maize inbred lines. **(A)** Frequency distribution of first-order branch number of tassel. **(B)** Frequency distribution of ear height. **(C)** Frequency distribution of spindle length of tassel. **(D)** Frequency distribution of plant height. **(E)** Frequency distribution of ear height/plant height ratio.

We selected 160 maize inbred lines from Jinzhong and Xinzhou regions for testing their plant height, ear height, spindle length of tassel and first-order branch number of tassel characteristics. The maximum first-order branch number of tassel among the 160 maize inbred lines in Jinzhong and Xinzhou was 24 and 23, respectively, with an average of 6.29 and 6.33 per line. Notably, there were also inbred lines identified in both regions that lacked any first-order branch number of tassel (Figure 1A; Supplementary Table S2). The average ear height of 160 maize samples from Jinzhong and Xinzhou regions was 79 and 80 cm, respectively. The frequency distribution of ear height traits in maize inbred lines in Xinzhou region showed a large fluctuation (Figure 1B; Supplementary Table S2). The phenotypic analysis of 160 maize inbred lines from Jinzhong and Xinzhou showed that the maximum and minimum values of the spindle length of tassel in Jinzhong were 44.47 and 19.87 cm, respectively, while the maximum and minimum values of the spindle length of tassel in Xinzhou were 44.08 and 19.65 cm, respectively. There was no significant difference in the average length of the spindle length of tassel between the two regions (Figure 1C; Supplementary Table S2). The maximum plant heights of 160 maize inbred lines in Jinzhong and Xinzhou reached 279 and 272 cm, respectively, while the minimum heights were 93 and 101 cm, respectively. Similarly to the changes in ear height, the frequency distribution of plant height in maize inbred lines showed comparable variations, with a more pronounced fluctuation observed in Xinzhou (Figure 1D; Supplementary Table S2). The maximum ear height/plant height ratios of maize inbred lines in Jinzhong and Xinzhou regions peaked at 0.52 and 0.53, respectively, while the minimum ratios stood at 0.24 and 0.25, respectively. The average ear height/plant height ratio in Jinzhong was slightly lower than that in Xinzhou. Interestingly, both regions exhibited a gap in the frequency distribution at 0.43, and there was a notable fluctuation in the frequency distribution within the range of 0.34–0.35 (Figure 1E).

### Chip data of affymetrix axiom and SNP mining of maize inbred lines

The average sample typing missing rate of the 160 sample raw data was 1.28% of the total number of missing loci, and the sample typing missing rate used was controlled within 4% (Supplementary Figure S1). The Affymetrix Axiom chip data had an average dish QC (DQC) value of 0.88, ranging from a maximum of 0.94 to a minimum of 0.83. Notably, over 95% of the high-quality probes met the QC detection criteria, with an average QC heterozygosity rate of 1.55%. Furthermore, the average detection rate across all loci in the samples was 99.29%, ensuring a sample quality control pass rate of 98.95%. The sample heterozygosity rate stood at 1.95% (Supplementary Table S3).

**FIGURE 2:**
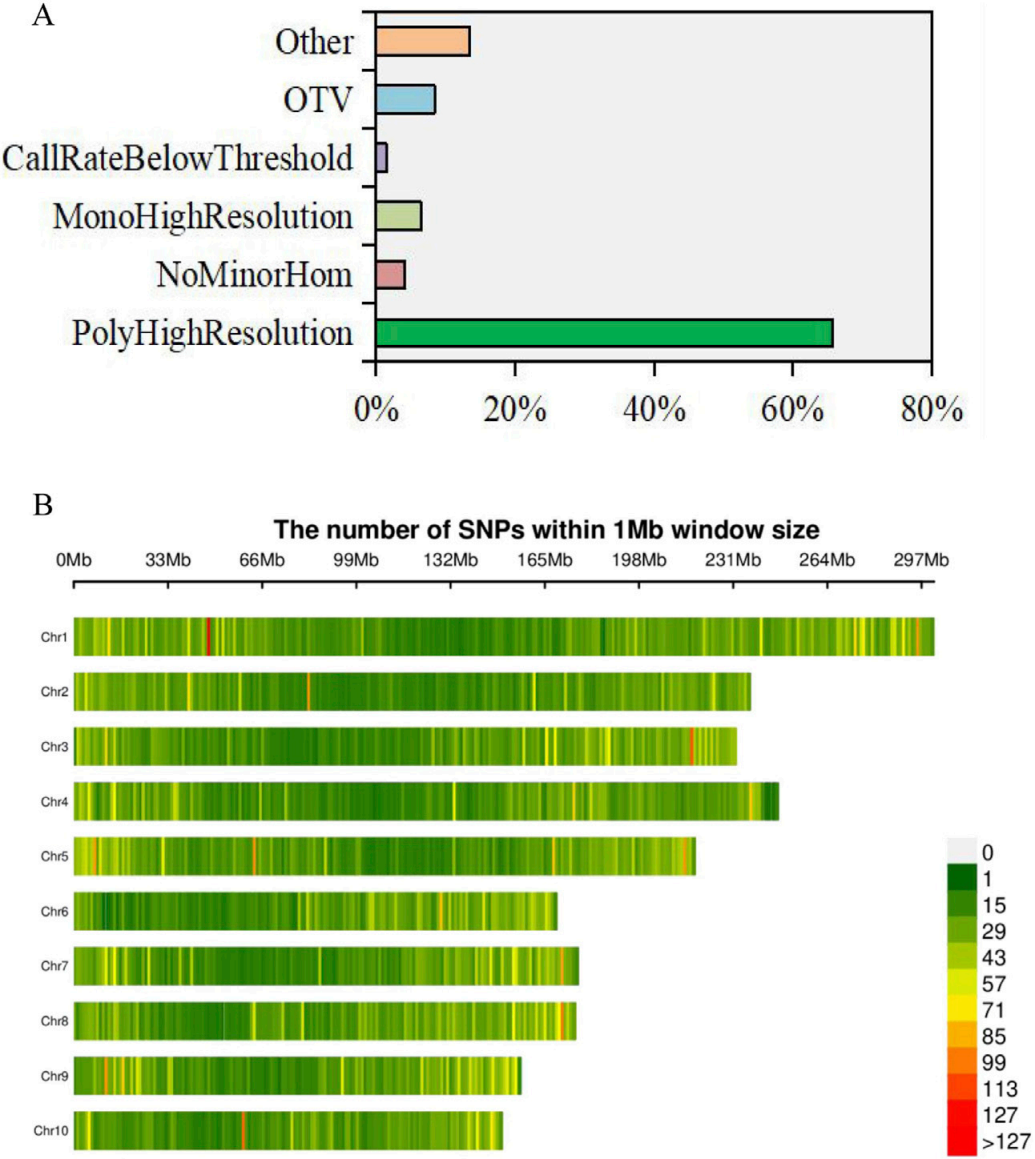
Chip data of affymetrix axiom and SNP mining of maize inbred lines. **(A)** Percentage of six typing types in total probe loci. **(B)** Distribution of SNPs on 10 maize chromosomes.

A total of 55,229 SNPs were retrieved from the Affy_SNPs database for the 160 sample loci probes, with an average typing success rate of 99.11%. The lowest typing success rate was 41.05% for probe locus: AX-91594479. Among all the probes, the average number of minor alleles was 150.7, the number of clusters was 2.7, the number of genotypes AA was 157.7, the number of genotypes AB was 12.5, and the number of genotypes BB was 206.3. The 36,300 probes previously exhibited the PolyHighResolution type, 2,305 probes exhibited the NoMinorHom type, 4,667 probes exhibited the OTV type, 3,635 probes exhibited the MonoHighResolution type, and only 858 probes exhibited the CallRateBelowThreshold type. Additionally, 13.5% of all probes belonged to other types (Figure 2A). After rigorous filtering, a total of 42,240 high-quality SNPs were identified. As depicted in Figure 2B, these SNPs were unevenly distributed across the 10 chromosomes of maize. Chromosome 1 hosts the highest number of SNPs, constituting 14.31% of the total, followed closely by chromosome 3 with 10.47%. Chromosomes 4 and 5 also contributed significantly, each accounting for over 10% of the total SNPs. Conversely, chromosome 10 has the lowest distribution of SNPs, accounting for just 6.51% of the total (Figure 2B; Supplementary Figure S2).

### Population structure of maize inbred lines

**FIGURE 3:**
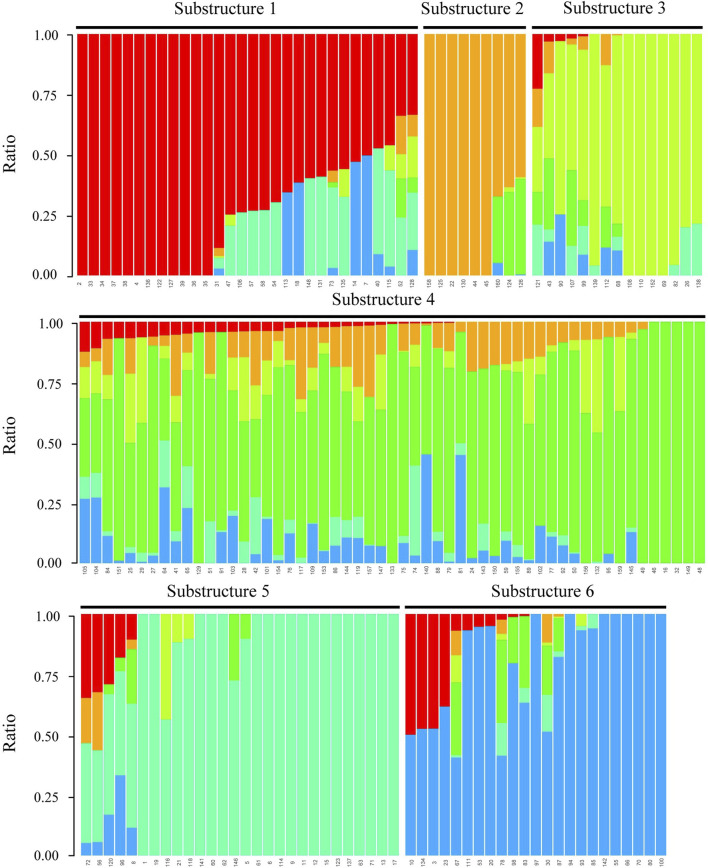
160 maize population structures based on SNP markers. Each color in the figure represented a substructure. The color scale represented the proportion of the sample’s genotype composition.

The optimal clustering number K = 6 was determined based on the minimal cross-validation error value achieved at this point, with subsequent variations remaining stable (Supplementary Figure S3). Consequently, 160 maize inbred lines were classified into six distinct substructures: Substructure 1, Substructure 2, Substructure 3, Substructure 4, Substructure 5, and Substructure 6 (Figure 3). The improved Substructure 4 group, represented by YE478 and ZHENG58, boasts the highest number of maize inbred lines among the six subgroups, totaling 55 inbred line materials. The Substructure 5 group comprised 28 maize inbred lines, notable examples of which were PH6WC and XY686M. The Substructure 6 group consisted of 23 maize inbred lines, featuring D1M, D3M, and 38P05M. The Substructure 3 group encompassed 15 maize inbred lines, with AMD5 and AMD293 among its past representatives (Figure 3; Supplementary Table S4).

### Nucleic acid diversity and genetic relationship of maize inbred lines

In order to study the nucleic acid diversity of maize subgroups, the π value of nucleic acid diversity was calculated for each subgroup. The Substructure 5 (π = 1.23 × 10^−4^), Substructure 1 (π = 1.25 × 10^−4^), and Substructure 2 (π = 1.50 × 10^−4^) displayed notably lower average π values, suggesting a reduced nucleic acid diversity potentially due to stronger selection pressure from natural selection or other factors. The Substructure 4 exhibited a higher π value of 2.44 × 10^−4^, indicating a reduced likelihood of it being subjected to significant selection pressure from natural selection or other external factors (Supplementary Figure S4). A total of 79,946 allelic variation loci were identified across 160 maize inbred lines, utilizing 42,240 high-quality SNPs. The comparison of genetic allele frequencies among taxa revealed the largest divergence between the Substructure 6 and the Substructure 5, while the Substructure 3 and Substructure 2 exhibited the smallest difference in allele frequencies (Table 1; Supplementary Figure S5). The six subgroups exhibited an average gene diversity of 0.356 and a mean polymorphism information content of 0.245. The genetic distances between these subgroups ranged from 0.06 to 0.38, while the genetic differentiation index varied from 0.83 to 0.85, averaging at 0.59.

**TABLE 1:** Genetic distances as measured by Nei’s distance (top diagonal) and pairwise Fst comparisons (bottom diagonal) between maize inbred groups.

Groups	Substructure 1	Substructure 2	Substructure 3	Substructure 4	Substructure 5	Substructure 6
Substructure 1	1	0.2145	0.3128	0.2701	0.1991	0.1755
Substructure 2	0.2145	1	0.3468	0.2975	0.2352	0.1967
Substructure 3	0.3128	0.3468	1	0.2145	0.214	0.1742
Substructure 4	0.2701	0.2975	0.2145	1	0.1905	0.1406
Substructure 5	0.1991	0.2352	0.214	0.1905	1	0.0812
Substructure 6	0.1755	0.1967	0.1742	0.1406	0.0812	1

An analysis of the genetic relationships and phylogenetic development among 160 maize inbred lines, utilizing SNP markers, revealed that maize within the same subpopulation exhibited closer genetic ties. The phylogenetic analysis categorized the 160 maize inbred lines into six distinct branches, mirroring closely the subgroup division obtained from the population structure analysis (Supplementary Figure S5; Supplementary Material S1). The Substructure 1 and Substructure 5 exhibited a close phylogenetic proximity, indicating a relatively frequent exchange of genetic material. The Substructure 6 exhibited the greatest phylogenetic distance from the other subgroups, suggesting limited gene flow between them. Only a few Substructure 6 shared close relationships with the Substructure 1 and Substructure 5. The gene exchange between various subgroups provided important basic materials for the improvement of maize inbred lines.

### Genome-wide association of plant type-related traits in maize

**FIGURE 4:**
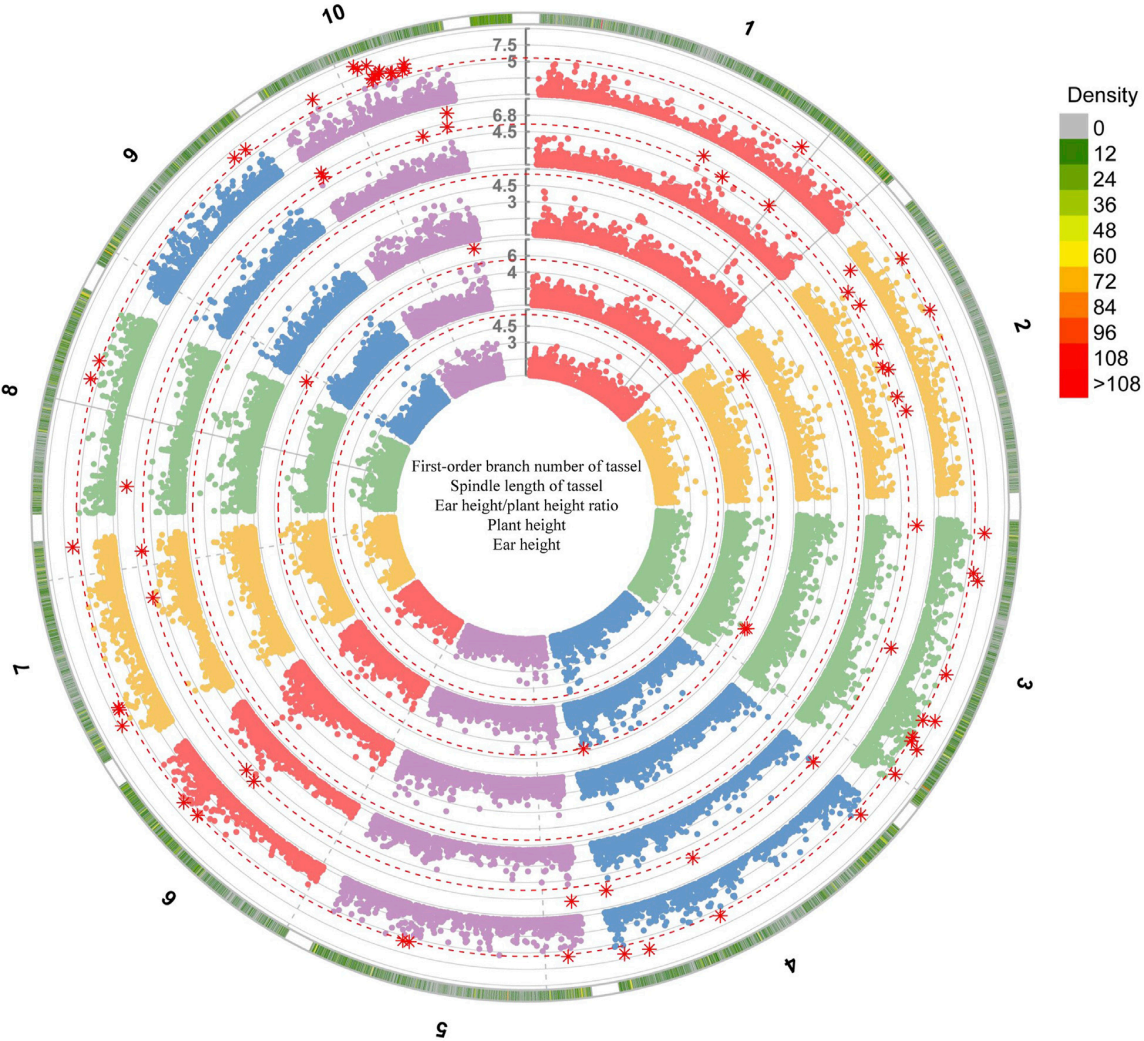
Genome-wide association analysis of plant type-related traits in Jinzhong based on the GLM model. The numbers represented the chromosome numbers of maize. The genome-wide association analysis of first-order branch number of tassel, spindle length of tassel, ear height/plant height ratio, plant height and ear height traits was conducted from the outside to the inside.

**FIGURE 5:**
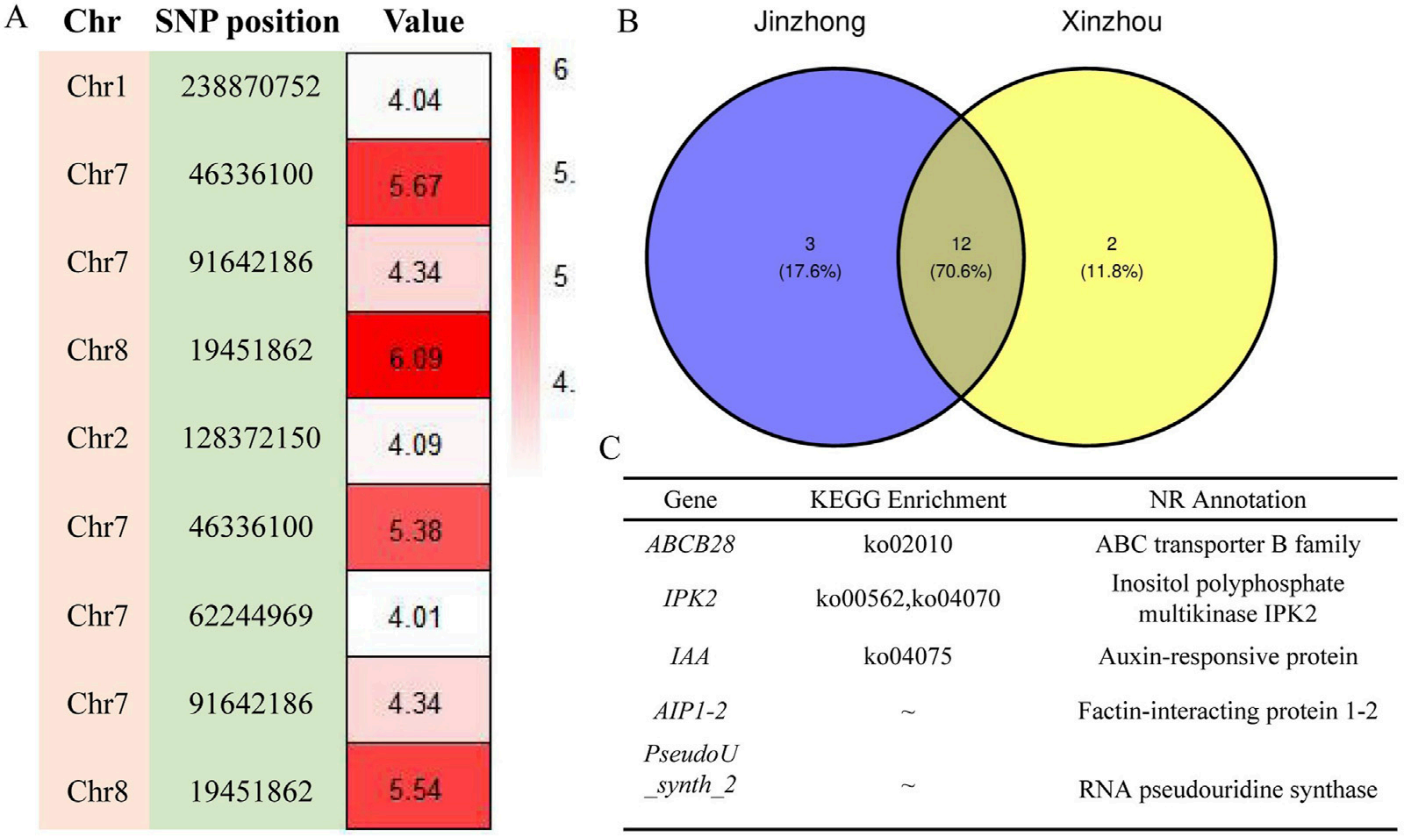
Genome-wide association analysis of first-order branch number of tassel trait for annotation of loci-related genes. **(A)** Analysis of the location and significance of GWAS association loci. The value displayed was −log_10_ (*P*-value). **(B)** Venn diagram of genes related to first-order branch number of tassel of maize inbred lines. **(C)** Functional annotation of part genes related to the first-order branch number of tassel in maize inbred lines from Jinzhong and Xinzhou.

Among the seven models selected for genome-wide association analysis, the GLM model of GWAS was the most suitable for maize first-order branch number of tassel and plant height traits, while the SUPER model was the most suitable for spindle length of tassel, ear height/plant height ratio and ear height traits (Figure 4; Supplementary Figures S9–S13). The GWAS between the first-order branch number of tassel and SNPs in 160 maize samples from Jinzhong and Xinzhou revealed no discernible systematic bias (Figure 4; Supplementary Figure S10). The GWAS of the first-order branch number of tassel in the Jinzhong region revealed four significant loci, primarily located on chromosomes 1, 7, and 8 (Figure 5; Supplementary Table S5). The GWAS analysis for the first-order branch number of tassel in Xinzhou region yielded five significant loci, predominantly located on chromosomes 2, 7, and 8 (Supplementary Table S5). Maize from Jinzhong and Xinzhou shared three loci significantly associated with the first-order branch number of tassel. The GWAS in Jinzhong identified 15 significant genes associated with 4 loci, while the analysis in Xinzhou revealed 14 significant genes linked to 5 loci in maize inbred lines. A total of 12 genes related to the first-order branch number of tassel were commonly associated in both Jinzhong and Xinzhou inbred lines, comprising 70.6% of the relevant genomic region (Figure 5). The molecular functional annotation and enrichment results showed that the UGT75L6 protein had transferase activity, the IPK2 protein had kinase activity, the AIP1-2 protein had protein binding activity, and the ABCB28 protein had ATP binding, TP enzyme activity, and ATPase activity, respectively. At the same time, ABCB28 was involved in transmembrane transport and IPK2 was involved in the biological process of phosphoinositide biosynthesis (Supplementary Table S6).

The GWAS of the spindle length of tassel and SNPs across 160 maize inbred lines from Jinzhong and Xinzhou did not exhibit any discernible systematic deviation (Supplementary Figures S12, S14). The GWAS analysis of the spindle length of tassel in Jinzhong region identified 14 significant associated loci, mainly distributed on chromosomes 2, 4, 5, 7, and 10, with 42.9% of the significant associated loci distributed on chromosome 2 (Supplementary Table S5). GWAS analysis of the spindle length of tassel in Xinzhou region revealed nine significant association loci, mainly distributed on chromosomes 2, 5, 7, and 10. All significant association loci in Xinzhou region were included in the significant association loci in Jinzhong region (Supplementary Table S5). The GWAS in Jinzhong region revealed 55 significant genes associated with 14 loci, while the analysis in Xinzhou yielded 35 significant genes linked to 9 loci in maize inbred lines. A total of 35 genes were commonly associated with the spindle length of tassel in both Jinzhong and Xinzhou inbred lines, comprising 63.6% of the relevant genomic region (Supplementary Figure S14; Supplementary Table S6). Among the shared genes between Jinzhong and Xinzhou, 88.6% of the expressed proteins had annotations in the Swiss-Prot database, while 82.9% of the gene names had been confirmed. At the same time, these genes were functionally annotated and enriched, such as *ERD7* gene involved in endocytosis pathway, *CML32* gene involved in plant-pathogen interaction, *GLU3* gene involved in starch and sucrose metabolism, and *ATG10* gene involved in autophagy regulation (Supplementary Table S6).

The GWAS association analysis of ear height trait in maize inbred lines in Jinzhong region obtained 13 significant loci, mainly distributed on chromosomes 1, 3, 4, 5, 7, 8 and 10 (Supplementary Figures S12, S14; Supplementary Table S5). The GWAS analysis for ear height in maize inbred lines in Xinzhou region revealed eight significant loci, predominantly found on chromosomes 1, 3, and 4 (Supplementary Table S5). Based on the candidate regions 100 kb upstream and downstream of the significant loci, 57 significantly associated genes were obtained for 13 loci significantly associated with maize inbred lines in Jinzhong region, and 52 significantly associated genes were obtained for 8 loci significantly associated with maize inbred lines in Xinzhou region. A total of 27 genes are commonly associated with the ear height trait in maize inbred lines from both Jinzhong and Xinzhou regions (Supplementary Figure S5; Supplementary Table S6). The functions of genes related to the ear height trait were annotated and enriched. The results showed that the *PBS1* gene was enriched in the plant-pathogen interaction pathway, and the *IAA4* gene was enriched in the auxin signaling pathway of the plant hormone signaling pathway (Supplementary Table S6).

The GWAS analysis of plant height traits in maize inbred lines in Jinzhong region identified five significant loci, mainly distributed on chromosomes 2, 3, 7, and 9 (Supplementary Figures S12, S14; Supplementary Table S5). The GWAS analysis of plant height traits in maize inbred lines in Xinzhou region identified three significant loci, mainly distributed on chromosomes 3 and 7 (Supplementary Table S5). Significant association loci were detected at 169,704,259 bp on chromosome 3 and 95581049 bp on chromosome 7 in the maize inbred lines from Jinzhong and Xinzhou, respectively (Supplementary Table S5). The GWAS in Jinzhong identified 26 significant genes associated with 5 loci, while the analysis in Xinzhou revealed 3 significant genes linked to 3 loci in maize inbred lines. Notably, all significant genes in Xinzhou were encompassed within those identified in Jinzhong (Supplementary Figure S15). We used multiple databases to compare and annotate the genes associated with plant height traits in maize inbred lines from Jinzhong and Xinzhou, which were annotated as BT2 (NM_001136739.1) and (ICMT NM_001365697.1 and XM_008674752.3) genes, respectively (Supplementary Table S6).

Based on the phenotypic data of ear height and plant height, we further conducted a genome-wide association analysis of the ratio of ear height to plant height. Compared with ear height and plant height traits, GWAS of ear height/plant height ratio obtained more SNPs. Among them, 23 significant loci were obtained by GWAS analysis of ear height/plant height ratio of maize inbred lines in Jinzhong region, mainly distributed on chromosomes 1, 2, 3, 4, 5, 8, 9 and 10 (Supplementary Figures S12, S14; Supplementary Table S5). The GWAS analysis of the ear height/plant height ratio trait of maize inbred lines in Xinzhou region identified 17 significant loci, primarily located on chromosomes 1, 3, 5, 6, 8, and 10 (Supplementary Figures S12, S14; Supplementary Table S5). At the same time, high-density loci were identified on chromosome 5 within the range of 24.50 Mb–24.72 Mb in the maize inbred lines from Jinzhong and Xinzhou. The GWAS in Jinzhong identified 136 significant genes associated with 23 loci, whereas the analysis in Xinzhou revealed 106 significant genes linked to 17 loci in maize inbred lines. Between the regions of Jinzhong and Xinzhou, 89 genes were commonly associated with the ratio of ear height to plant height in maize inbred lines, comprising 58.2% of the total identified genes (Supplementary Figure S15; Supplementary Table S6). The 89 shared genes between Jinzhong and Xinzhou regions were enriched in seven distinct pathways, including glutathione metabolism, metabolism of xenobiotics by cytochrome P450, drug metabolism - cytochrome P450, carotenoid biosynthesis, protein processing in endoplasmic reticulum, ribosome biogenesis in eukaryotes, plant hormone signal transduction (Supplementary Table S6).

### Potential key genes of maize type-related traits

**FIGURE 6:**
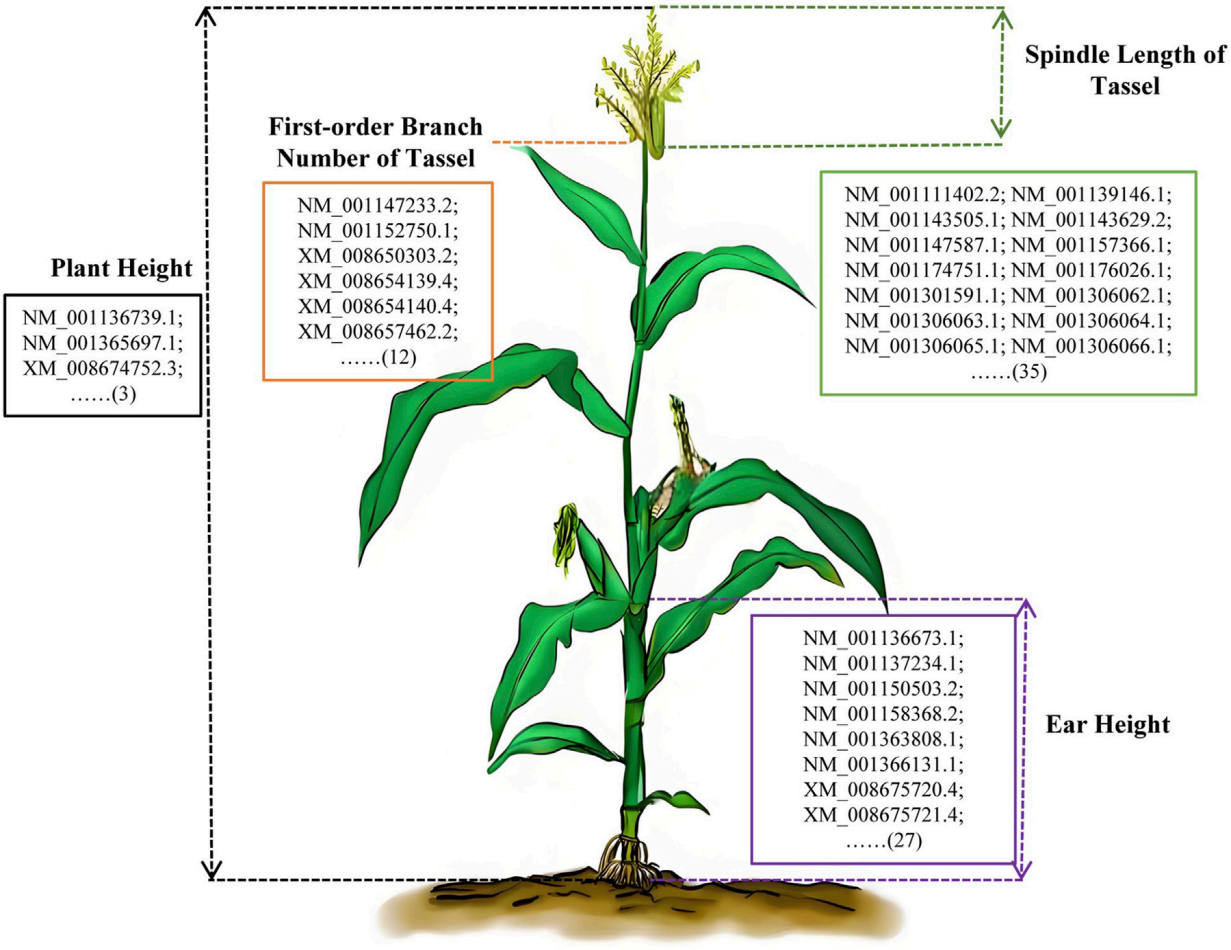
Potential key genes of maize type-related traits. The different colored lines represented the schematic diagram of the measured phenotype. Orange line, first-order branch number of tassel; Green line, spindle length of tassel; Black line, plant height; Purple line, ear height. The number enclosed in parentheses signifies the total count of potential key genes identified through genome-wide association analysis. The details of the genes abbreviated with an ellipsis could be found in Supplementary Table S6.

We identified 12, 35, 27, 3, and 89 genes that were significantly associated with first-order branch number of tassel, spindle length of tassel, ear height, plant height, and ear height/plant height ratio, respectively. There were unannotated genes and alternative splicing of genes in these significantly associated genes of maize plant type (Figure 6; Supplementary Table S6). We screened and eliminated genes that were unannotated genes and alternative splicing. Finally, 6, 22, 14, 2, and 37 genes were identified as significantly associated with first-order branch number of tassel, spindle length of tassel, ear height, plant height, and ear height/plant height ratio, respectively (Supplementary Table S6). The identification of these genes not only sheds light on the genetic underpinnings of maize plant type traits but also serves as a valuable genetic resource for future biological breeding efforts. By understanding the specific functions and interactions of these genes, researchers can develop targeted breeding strategies to enhance desirable plant type traits in maize.

## Discussion

Maize germplasm resources constitute a vital foundation for molecular breeding research, improvement of maize inbred lines, and selection of superior new varieties. The abundance of high-quality germplasm resources ultimately dictates the success of maize breeding theories and practices. At present, there are more than 80,000 maize germplasm resources collected worldwide, including about 20,000 each from the International Maize and Wheat Improvement Center (CIMMYT) in Mexico and the United States Germplasm Bank (Ortiz et al., 2010). The first expansion plan for maize germplasm resources is completed in 2006, with 12,113 maize germplasm resources evaluated and a large number of excellent local germplasm with unique breeding value identified and discovered, including high yield, resistance to pests and diseases, drought resistance, and low nitrogen tolerance (Salhuana and Pollak, 2006). The 160 maize inbred line populations that were selected for this study encompassed a comprehensive representation, comprising both core maize germplasm inbreds and parental inbred lines of hybrids sourced from diverse ecological zones within Shanxi Province. The germplasm resources utilized in this study effectively integrated the common features of theoretical research, while simultaneously aiming to provide genetic resources to guide local breeding efforts. The selection of germplasm resources is crucial for analyzing specific traits through population genetics.

The manifestation of traits associated with maize plant type has a direct influence on overall maize yield. The male and female tassels of maize compete for photosynthetic products, with a larger male tassel inhibiting the accumulation of such products in the female tassel (Monneveux et al., 2008). The first-order branch number of tassel is negatively correlated with maize yield (Li et al., 2011). However, commercialized maize seed production requires a large tassel in the male parent to reduce seed production risks and costs. The spindle length of tassel exhibits a highly significant positive correlation with the maize stalk’s resistance to breaking, making it a crucial reference trait in selecting novel maize varieties that demonstrate robust resistance to stalk breakage (Zhang Fang-kui et al., 2006). Plant height and ear height, as important agronomic traits in maize plant type breeding, directly affect the efficiency of nutrient and light energy utilization in maize plants (Khush, 2001; Fernandez et al., 2009). The results of this study showed that the phenotypic diversity of the main maize germplasm resources was relatively rich. The ear height of 160 maize inbred line population materials was concentrated between 50 and 110 cm, and the plant height was mainly distributed between 150 and 250 cm, with a wide range of variation in ear height and plant height. Proper plant height and ear height can not only improve the efficiency of plant photosynthesis and the utilization of nitrogen and other nutrients in plants, but also rationally allocate the transportation and storage of nutrients, which is beneficial for the accumulation of photosynthetic products in the ear, thereby improving the biological yield of maize (Khush, 2001; Fernandez et al., 2009).

Maize germplasm boasts a vast array of genetic diversity, serving as a treasure trove of resources for enhancing desired traits in maize breeding programs (Warburton et al., 2008; Frascaroli et al., 2013). Maize inbred lines are the basic materials for molecular breeding and conventional breeding research, which help to improve the efficiency of maize breeding (Yu et al., 2007; Yue-Hua et al., 2007; Ali et al., 2011). A high-density genetic map of maize containing 556,809 SNPs is constructed using 513 maize inbred lines from tropical, subtropical, and temperate regions as experimental materials (Yang et al., 2014). Affymetrix Axiom^®^, a high-density maize genotyping chip, was utilized in this study, resulting in the identification of 55,229 SNP loci. The number of SNPs in maize inbred lines is closely related to the size of the population. A diverse HNAU-NAM1 population, encompassing 12 subgroups and totaling 1,625 lines, is meticulously crafted through hybridization, self-pollination, and backcrossing, utilizing 13 genetically distinct maize inbred lines sourced from various geographical locations (Yang et al., 2011). In this study, 160 maize inbred lines were divided into 6 subgroups. It can be seen that there is extensive genetic variation within different maize populations, which in turn leads to different population structures.

The strategy of mining SNPs and genes associated with important traits in plants using GWAS has been widely applied (Crowell et al., 2016; Anwer et al., 2018; Pang et al., 2021). In previous studies on maize plant height, a mixed linear model is used to associate the loci located on chromosome 3, and 83, 86, and 38 loci are identified in the 165.73–166.37 Mb region of chromosome 3, respectively (Yang et al., 2014; Pan et al., 2017). In this study, 5 and 3 significantly associated loci were identified in Jinzhong and Xinzhou, respectively, mainly distributed on chromosomes 3 and 7. The SNP location on chromosome 3 was similar to the chromosome location of the previous study on plant height mapping. Four loci significantly associated with ear height and two loci significantly associated with ear height/plant height ratio are identified on chromosomes 3, 5, 6, and 8 in 284 tropical, subtropical, and temperate maize accessions (Sun Qiang et al., 2022). In this study, five significant loci associated with ear height were identified on chromosome 3 in maize inbred lines from Jinzhong and Xinzhou. In addition, two loci associated with ear height were identified on chromosomes 1 and 4, and 14 loci significantly associated with the ratio of ear height to plant height were also identified. These results further indicate that these shared loci play an important role in regulating plant type-related traits in maize. However, due to the differences in research populations, the role of specific loci cannot be ignored.

## Conclusion

Plant type directly affects the photosynthetic efficiency, lodging resistance, and stress resistance of plants, which in turn affects crop yield and quality. In this study, 160 maize inbred lines were used as experimental materials, and phenotype data for five plant type related traits, plant height, ear height, spindle length of tassel and first-order branch number of tassel, were obtained, along with 42,240 high-quality SNPs. We identified 12, 35, 27, 3, and 89 genes that were significantly associated with first-order branch number of tassel, spindle length of tassel, ear height, plant height, and ear height/plant height ratio, respectively. The phenotypic data and population structure information of maize provided important data support for maize cultivation in Xinzhou and Jinzhong regions. At the same time, the significant associated genes of plant type-related traits provided theoretical basis and gene resources for breeding ideotype in maize.

## Data Availability

The datasets presented in this study can be found in online repositories. The data information is publicly accessible at the Figshare database (https://doi.org/10.6084/m9.figshare.26861719).
