# Supplementary material for: Molecular markers and molecular basis of plant type related traits in maize
Source: Front Genet. 2024 Nov 1;15:1487700. doi: 10.3389/fgene.2024.1487700 (PMC11564161; doi:10.3389/fgene.2024.1487700)
Supplement: Supplementary file 3 [file Table1.docx]

Supplementary Material

# 1 Supplementary Figures and Tables

## 1.1 Supplementary Figures


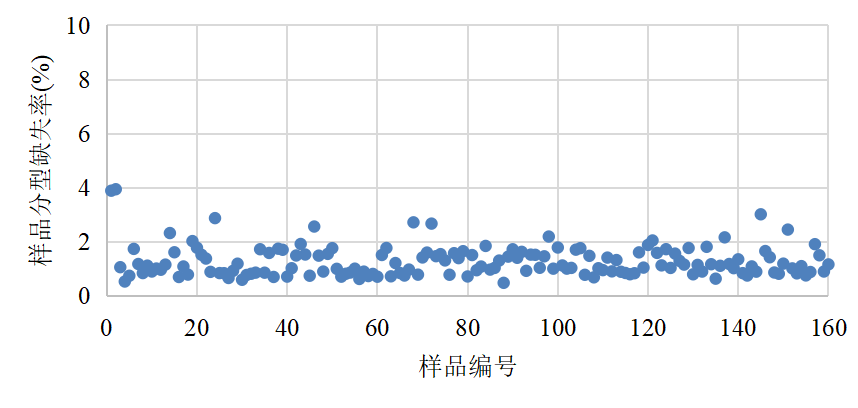


Figure S1. Sample typing deletion rate (missing loci/total loci).


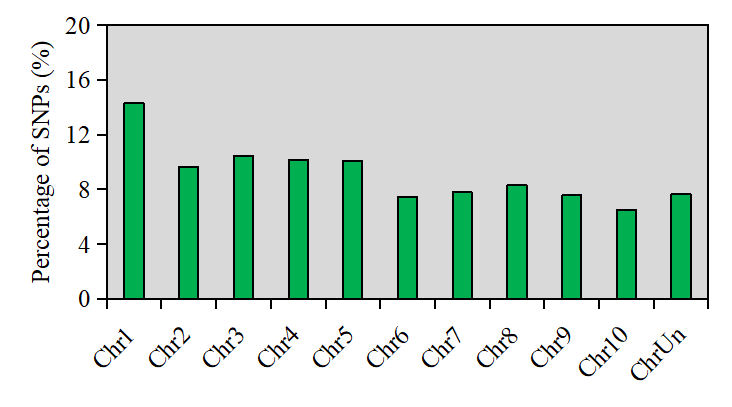


Figure S2. Percentage distribution of SNP sites on maize chromosomes.


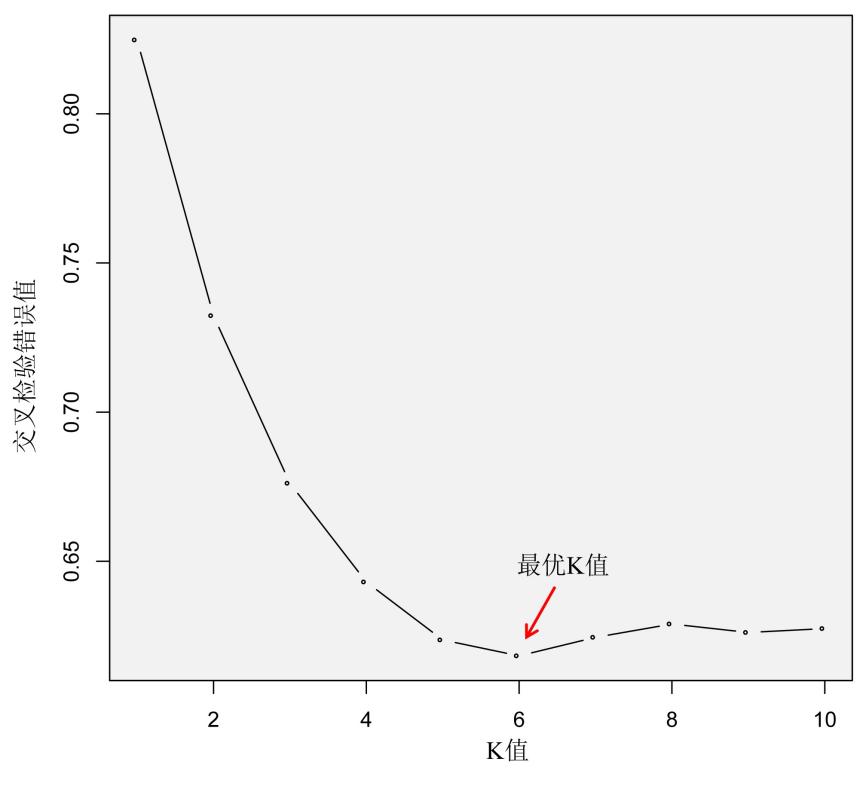


Figure S3. Cross-test error value versus K value.


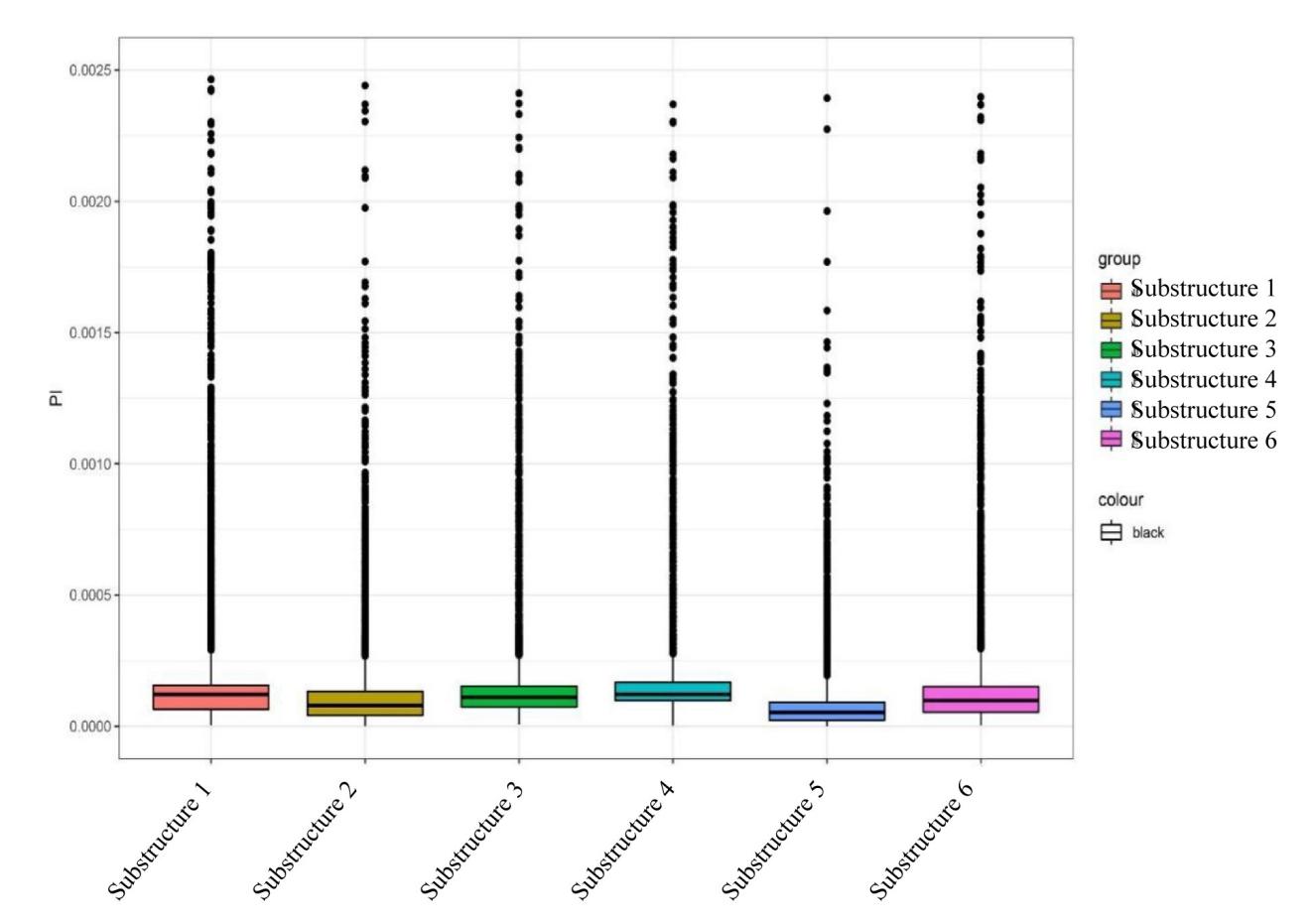


Figure S4. Nucleic acid diversity in 160 different subgroups of maize inbred lines.


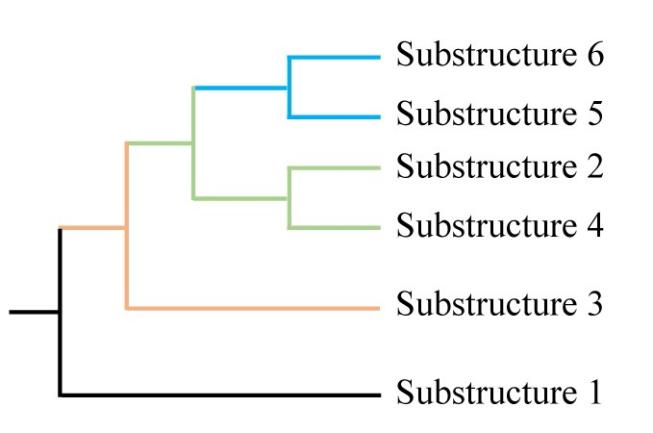


Figure S5. Phylogenetic relationships among subgroups of maize inbred lines


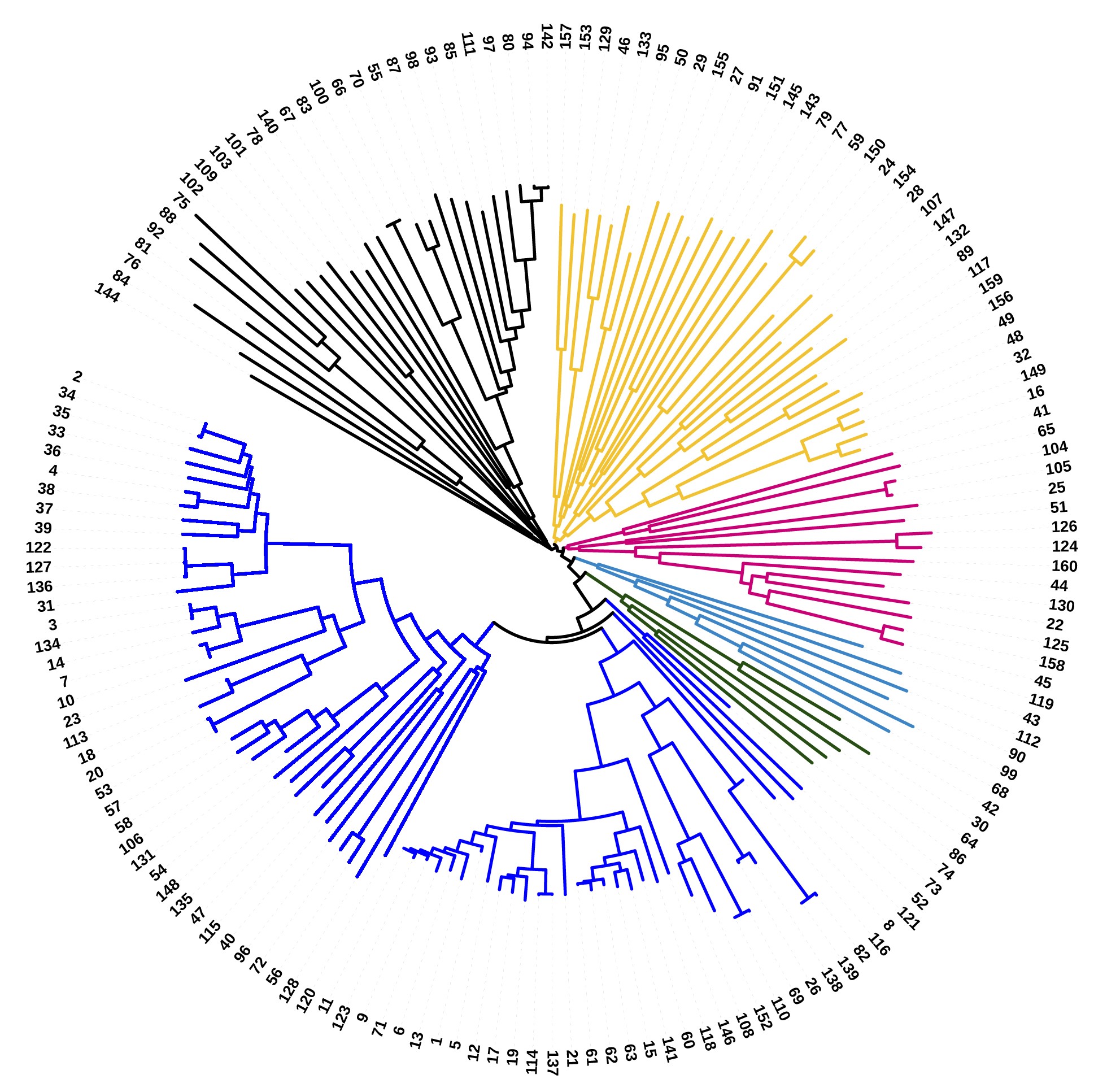


Figure S6. Phylogenetic tree of 160 maize inbred lines. The Arabic numerals indicated the ID of the maize inbred line. For detailed information, see table S1.


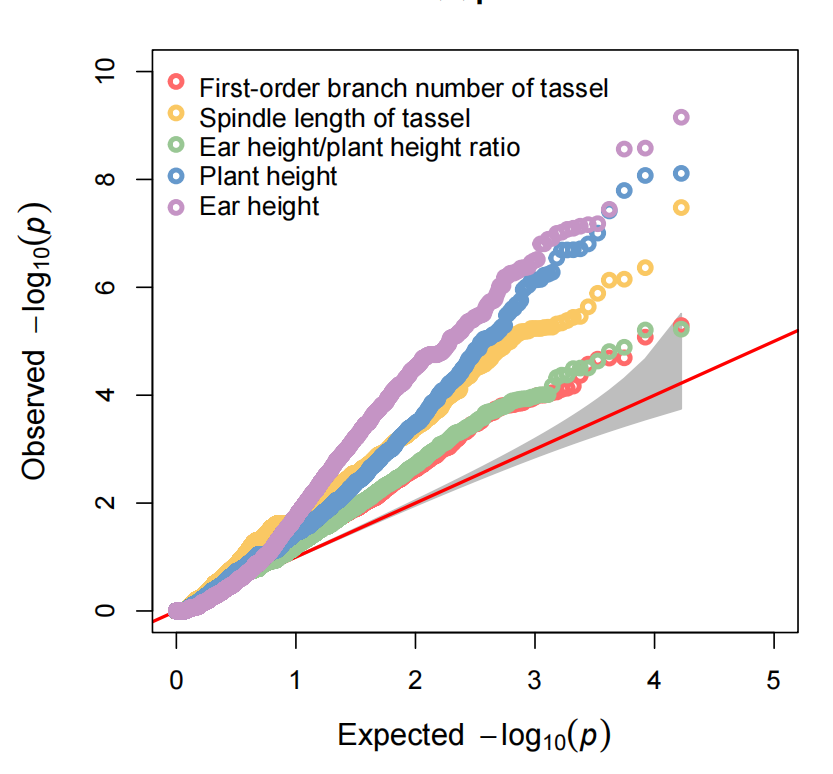


Figure S7. The Quantile-Quantil plot of genome-wide association of plant type-related traits in Jinzhong based on the GLM model.


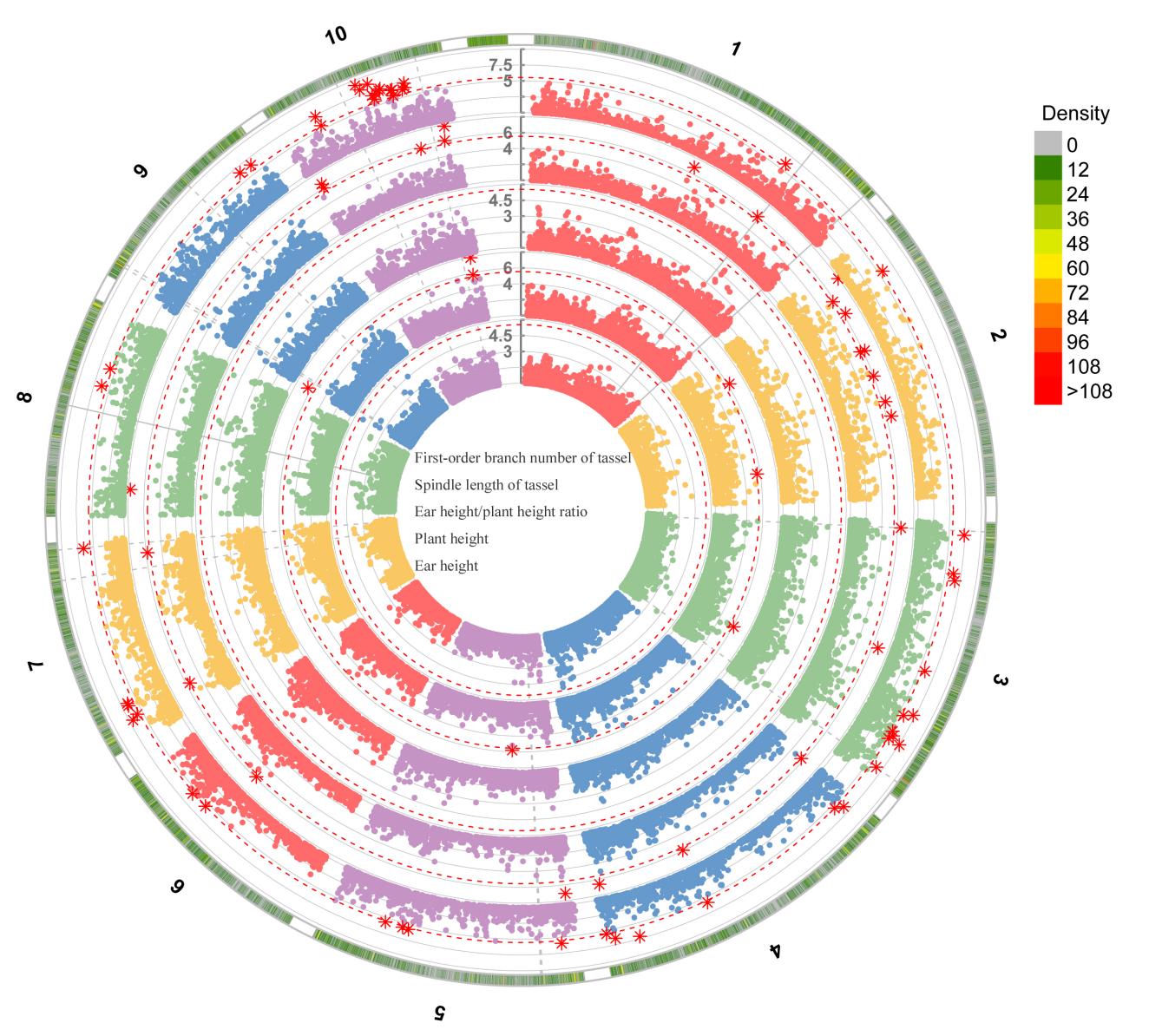


Figure S8 Genome-wide association analysis of plant type-related traits in Xinzhou based on the GLM model. The numbers represented the chromosome numbers of maize. The genome-wide association analysis of first-order branch number of tassel, spindle length of tassel, ear height/plant height ratio, plant height and ear height traits was conducted from the outside to the inside.


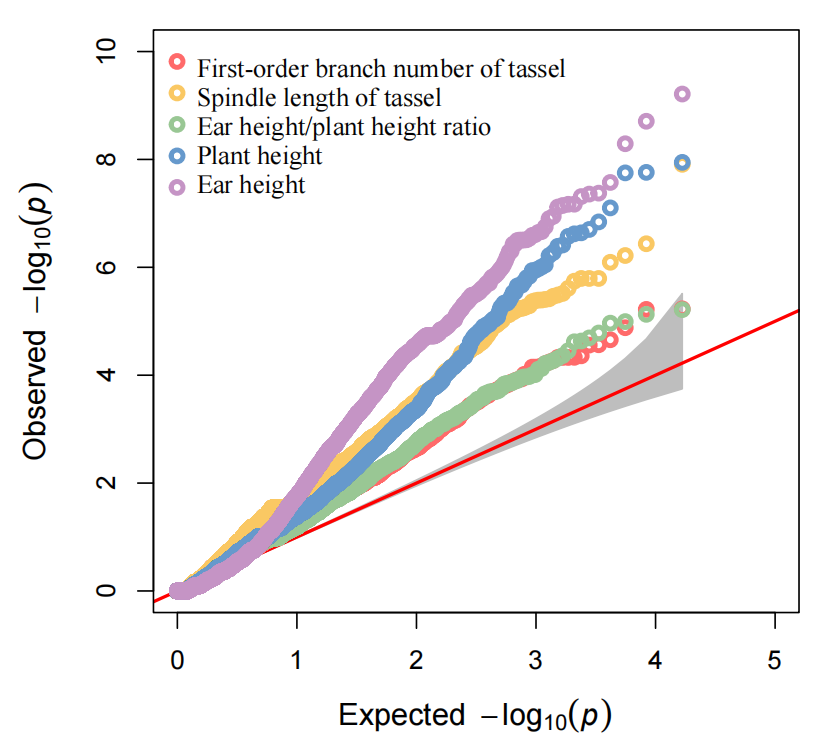


Figure S9. The Quantile-Quantil plot of genome-wide association of plant type-related traits in Xinzhou based on the GLM model.


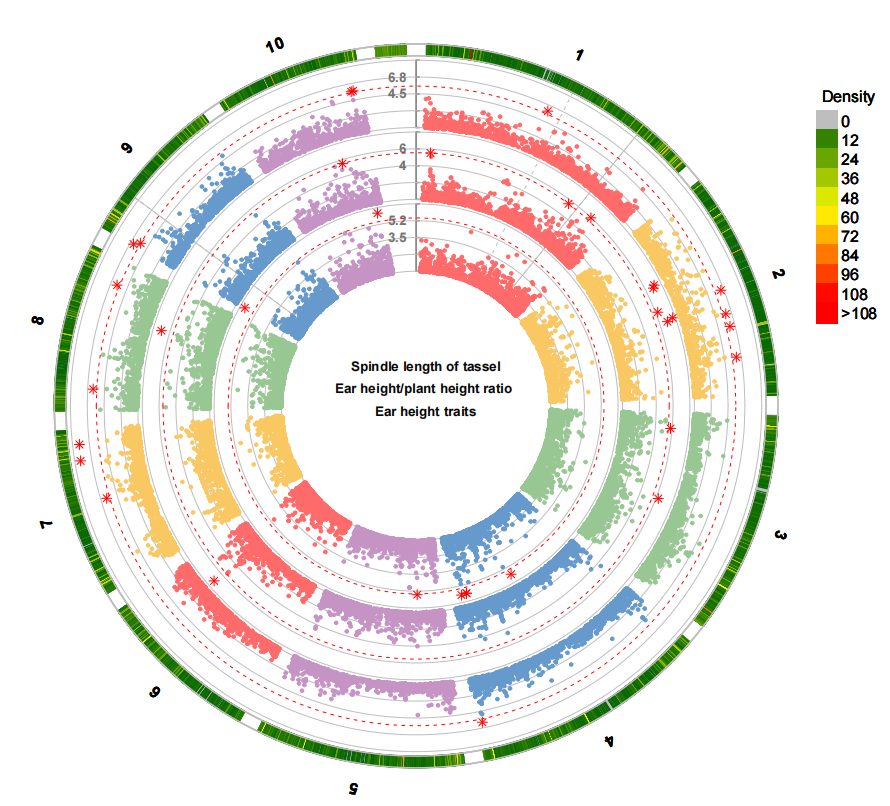


Figure S10 Genome-wide association analysis of plant type-related traits in Jinzhong based on the SUPER model. The numbers represented the chromosome numbers of maize. The genome-wide association analysis of spindle length of tassel, ear height/plant height ratio and ear height traits was conducted from the outside to the inside.


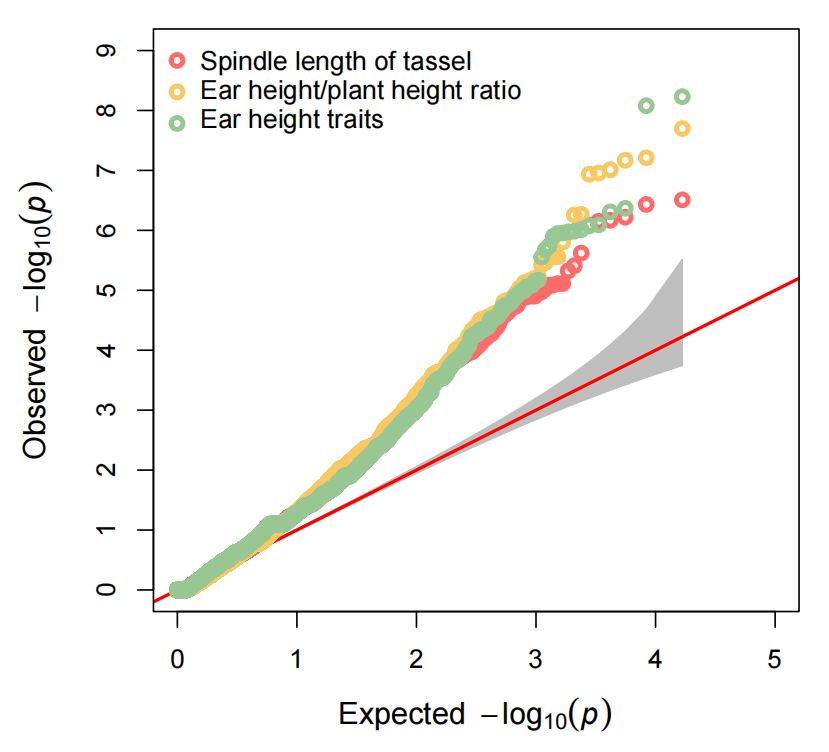


Figure S11. The Quantile-Quantil plot of genome-wide association of plant type-related traits in Jinzhong based on the SUPER model.


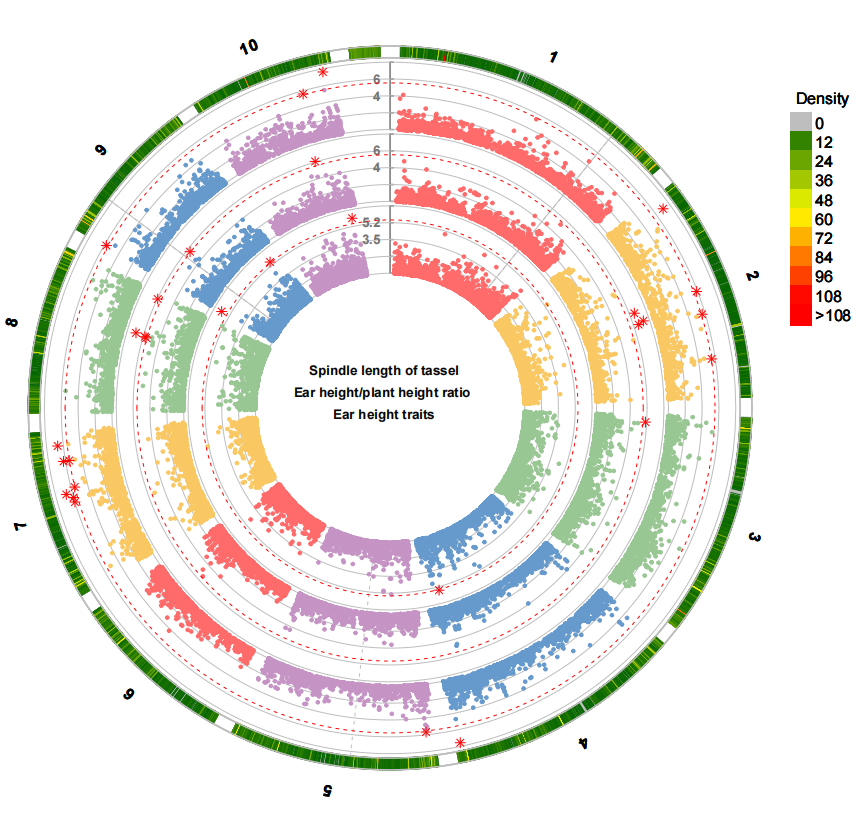


Figure S12. Genome-wide association analysis of plant type-related traits in Xinzhou based on the SUPER model. The numbers represented the chromosome numbers of maize. The genome-wide association analysis of spindle length of tassel, ear height/plant height ratio and ear height traits was conducted from the outside to the inside..


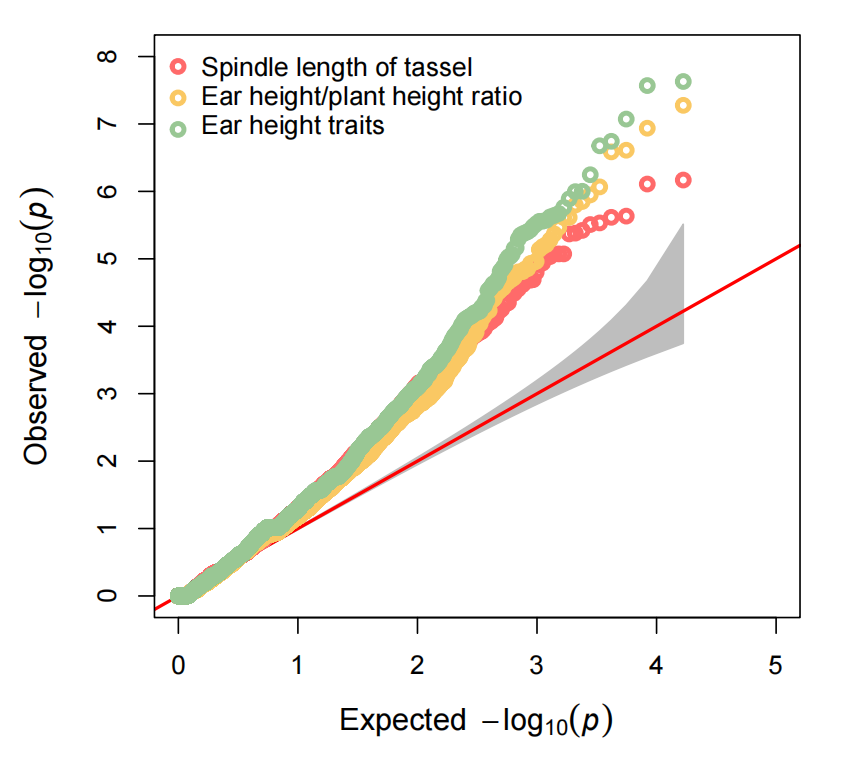


Figure S13. The Quantile-Quantil plot of genome-wide association of plant type-related traits in Xinzhou based on the SUPER model.


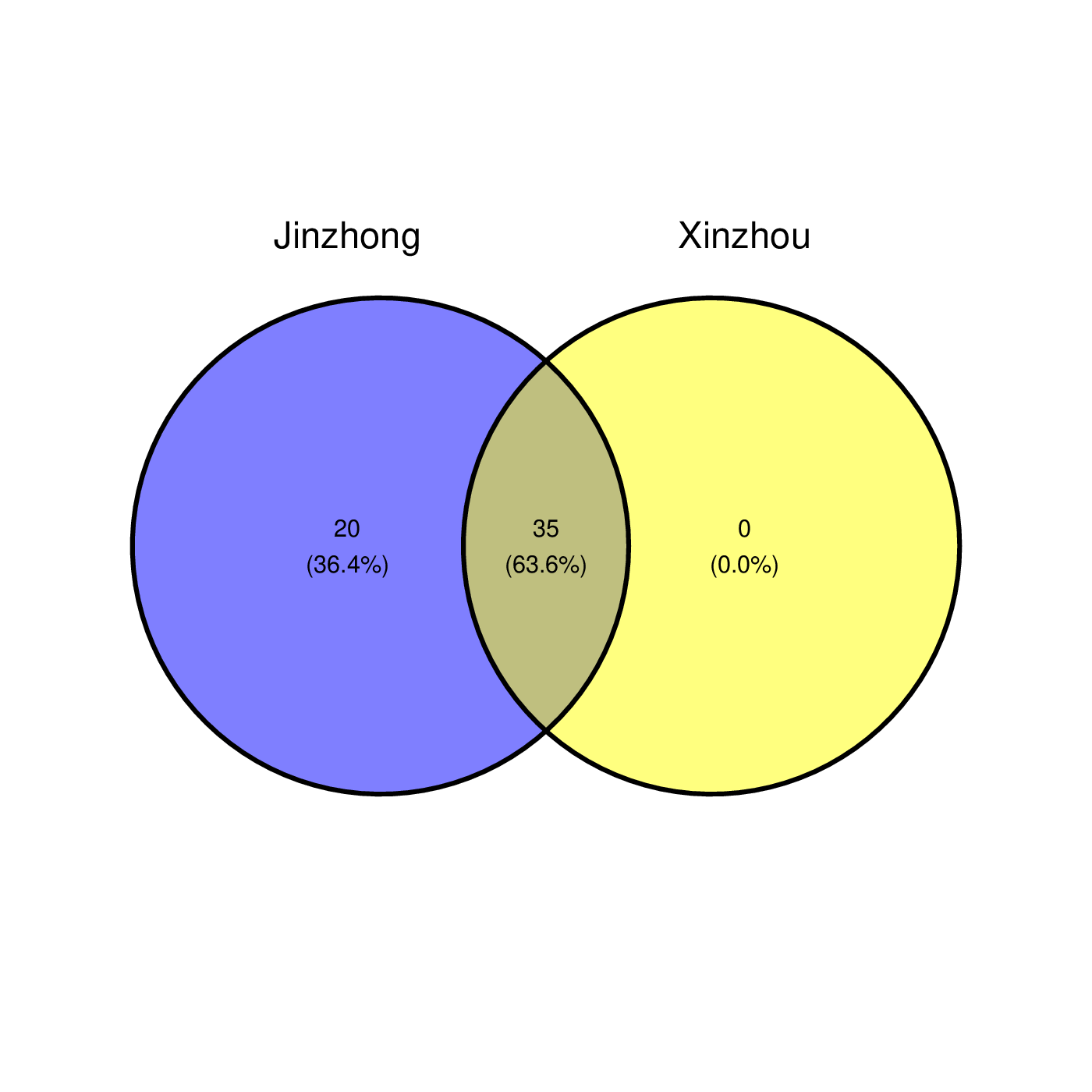


Figure S14. Venn diagram of genes related to tassel spindle traits in maize inbred lines from Jinzhong and Xinzhou.


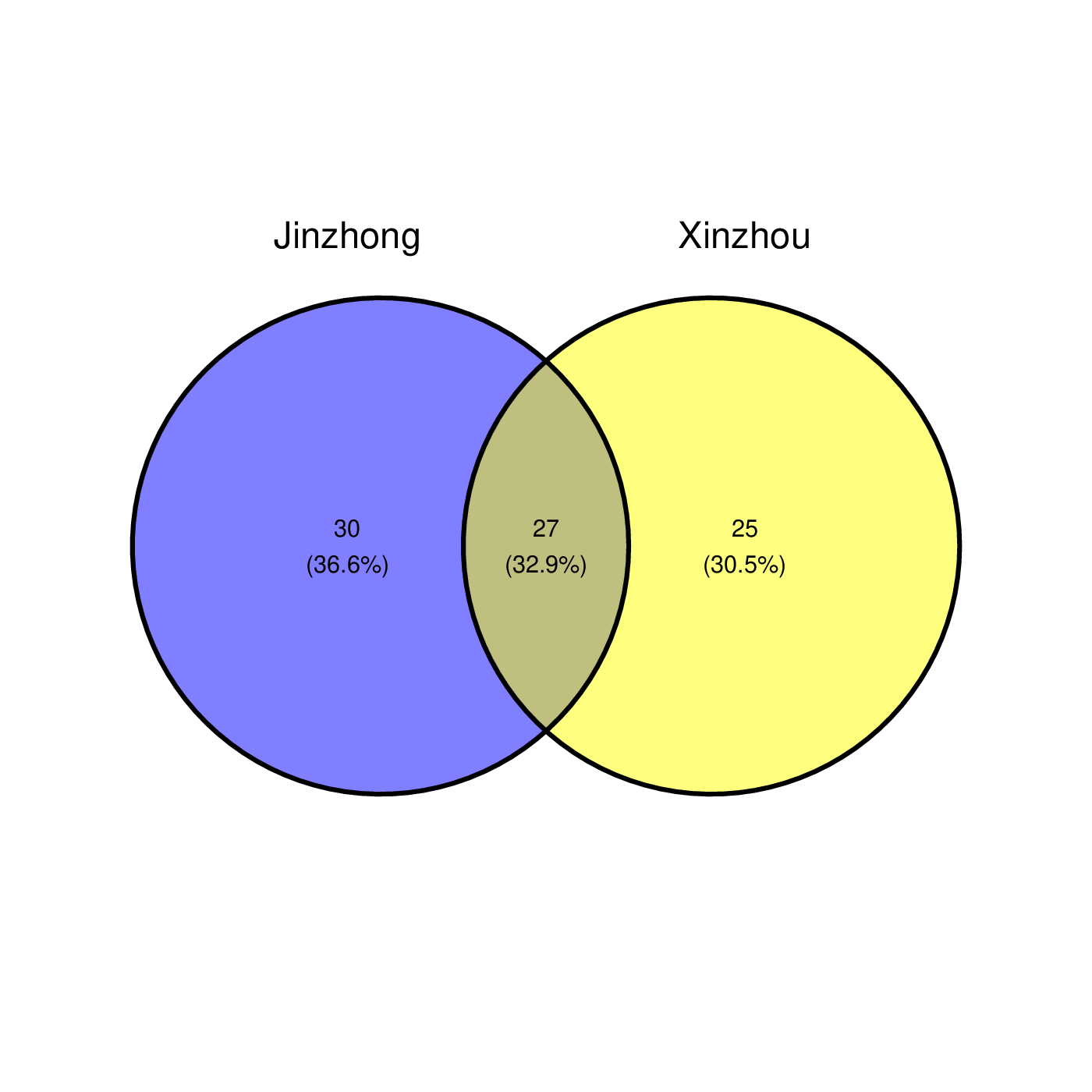


Figure S15. Venn diagram of genes related to ear height in maize inbred lines from Jinzhong and Xinzhou.


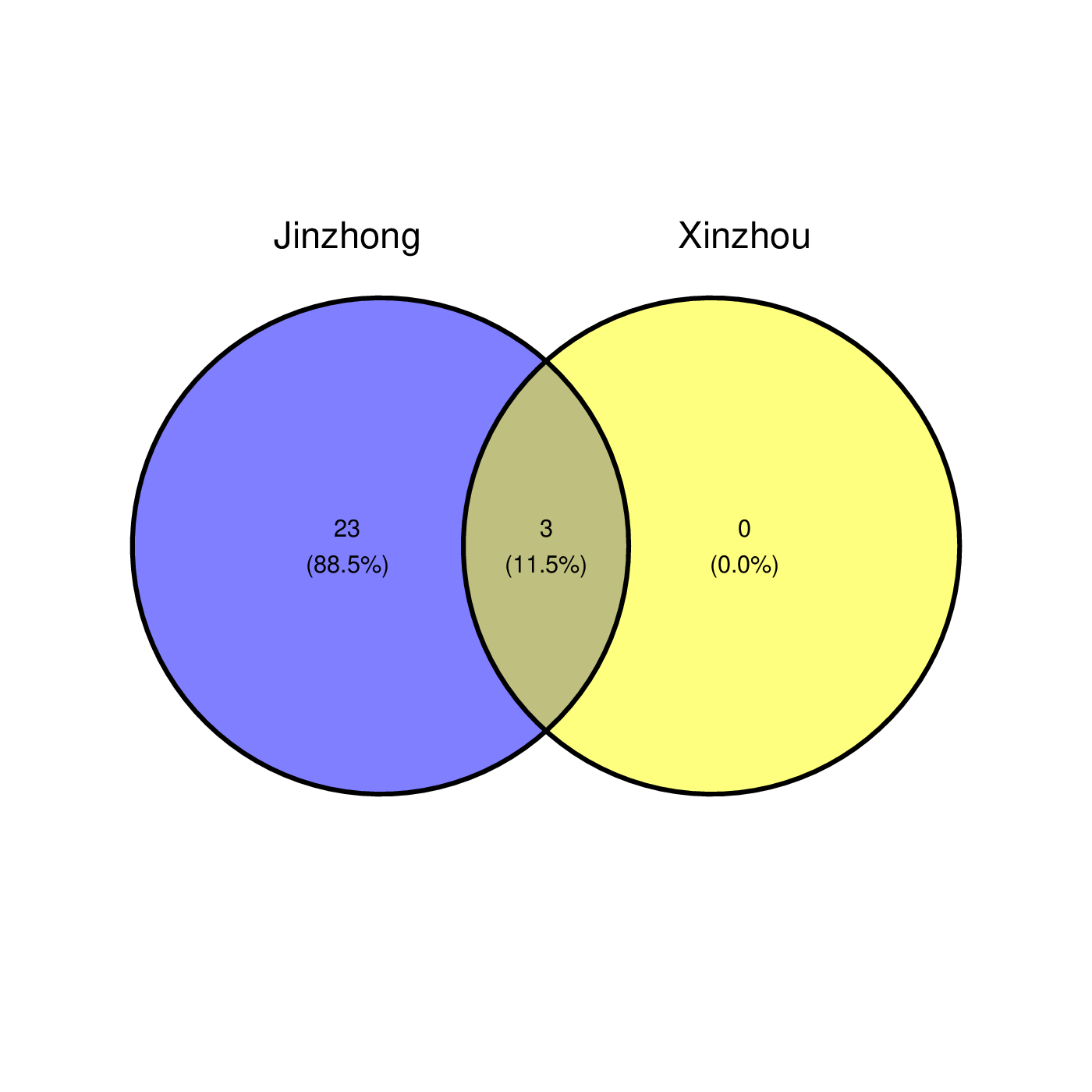


Figure S16. Venn diagram of genes related to plant height traits in maize inbred lines from Jinzhong and Xinzhou

## 1.2 Supplementary Tables

Table S1. Name/Source of the core inbred lines.

Table S2. Statistical analysis of phenotype data for 160 maize inbred lines.

Table S3. Raw data quality control statistics.

Table S4. Membership of 160 maize inbred lines corresponding to different subgroups.

Table S5. Genome-wide association significant SNP sites for plant type-related traits in maize.

Table S6. Functional annotation of genes related to the plant type-related traits in maize inbred lines from Jinzhong and Xinzhou.
